# Med9 Is a Novel eIF4E-Interacting Protein in *Saccharomyces cerevisiae*

**DOI:** 10.3390/ijms27156911

**Published:** 2026-08-01

**Authors:** Dora E. Vélez, Zorica Ristic, Daniela Ross-Kaschitza, Michael Altmann, Angélica Montiel-Dávalos, Vincent G. Osnaya, Yolanda Camacho-Villasana, Xochitl Pérez-Martínez, Greco Hernández

**Affiliations:** 1 mRNA and Cancer Laboratory, Subdirección de Investigación Básica, National Institute of Cancer (Instituto Nacional de Cancerología, INCan), Mexico City 14080, Mexico; dvelezuriza@gmail.com (D.E.V.); acilegnamontiel208@yahoo.com.mx (A.M.-D.); vgonzalez@inmegen.edu.mx (V.G.O.); 2 Institut für Biochemie und Molekulare Medizin (IBMM), Universität Bern, 3012 Bern, Switzerland; zorica.ristic@epic.ethz.ch (Z.R.); daniela.ross@unibe.ch (D.R.-K.); michael.altmann@unibe.ch (M.A.); 3 Cell Physiology Institute (Instituto de Fisiología Celular), Universidad Nacional Autónoma de México, Mexico City 04510, Mexico; ycamacho@ifc.unam.mx (Y.C.-V.); xperez@ifc.unam.mx (X.P.-M.)

**Keywords:** eIF4E, Med9, mRNA metabolism, translation initiation, carbon source

## Abstract

The cap-binding protein eukaryotic initiation factor (eIF) 4E is a key protein for mRNA metabolism. The biological role of eIF4E is defined by the specific protein it interacts with. Thus, eIF4E binds to a constellation of partners across eukaryotes. The best-characterized role of eIF4E is to promote mRNA translation through interaction with the translation factor eIF4G. To seek new interactors in the ascomycete *Saccharomyces cerevisiae*, we performed a genomic yeast two-hybrid screen using eIF4E as bait. In addition to the already reported p20 and Eap1, we identified Med9, a component of the RNA polymerase II Mediator complex. A physical interaction between eIF4E and Med9 was confirmed using recombinant proteins prepared in *E. coli* and further isolating the eIF4E–Med9 complex both by size-exclusion chromatography and by m^7^GTP-Sepharose pull-down experiments. Moreover, the in vivo interaction was also detected. Surprisingly, the eIF4E W75A mutation, which impairs binding to eIF4G, p20, and Eap1, only slightly affected the interaction with Med9 in the two-hybrid system. We further performed random mutagenesis to identify the Med9 amino acids involved in eIF4E recognition. Mutants F65A/I66A and F65A/I66A/H68N did not interact with eIF4E. Our data suggest that the eIF4E–Med9 complex may be formed in both the cytoplasm and the nucleus.

## 1. Introduction

The cap-binding protein eukaryotic initiation factor (eIF) 4E is a key protein for mRNA metabolism. The most characterized activity of eIF4E is its involvement in promoting mRNA translation [1]. Approximately 50 eIF4E-interacting proteins (4E-IPs) have been reported to bind to different eIF4E paralogs across diverse species to mediate mRNA export from the nucleus to cytoplasm, translation, or storage [2]. Each 4E-IP defines the process in which eIF4E gets involved. For example, when eIF4E makes a complex with eIF4G, it is able to drive translation initiation [3,4,5]; its interaction with the DEAD-box RNA helicase Rck/Xp54 enables eIF4E to mediate mRNA storage in cytoplasmic foci [6,7], and interaction with human homeodomain protein 9 (HOXA9) within the nucleus controls export of cyclin D1 and ornithine decarboxylase (ODC) mRNAs [8]. All eIF4E activities strictly depend on its association with 4E-IP; therefore, eIF4E behaves as a promiscuous and multifunctional wildcard at the crossroads between different RNA processes [2,9]. Thus, eIF4E is a crucial interface between gene expression and RNA metabolism.

The genes encoding eIF4E have undergone multiple duplications in many eukaryotes [2,10,11,12]. Fungal species contain one to seven paralogs of the *eIF4E* gene [12,13]. The ascomycete yeast *S. cerevisiae* possesses a single essential gene, *CDC33* encoding eIF4E [14,15], that promotes mRNA translation initiation [16,17]. *S. cerevisiae* eIF4E interacts with four proteins, namely eIF4G1, eIF4G2 [18,19,20], eIF4E-associated protein (Eap) 1 [20,21], and p20 [19,20,22,23]. eIF4E interaction with eIF4G1 or eIF4G2 drives mRNA translation [20,24,25,26]. Eap1 is involved in translation inhibition of specific mRNAs encoding nuclear pore membrane proteins [27] or in lipid synthesis [28,29]. Eap1 promotes translation during nitrogen deprivation [30] and also triggers degradation of a subset of mRNAs [31,32]. p20 modulates the translation of specific mRNAs by both eIF4E- and eIF4E-independent mechanisms [33,34,35]. Recently, it was shown that the eIF4E/p20 complex stimulates the translation of reporter mRNAs with variable 5′-UTRs in vitro [36].

Here, we report that Med9, a component of the RNA polymerase II transcriptional Mediator complex, is a novel partner of eIF4E and that their interaction might occur in the cytoplasm and the nucleus. We also show the eIF4E–Med9 complex forms in the phylogenetically close yeast *Saccharomyces kudriavzevii*.

## 2. Results

### 2.1. Med9 Is Novel eIF4E-Interacting Protein

**Figure 1:**
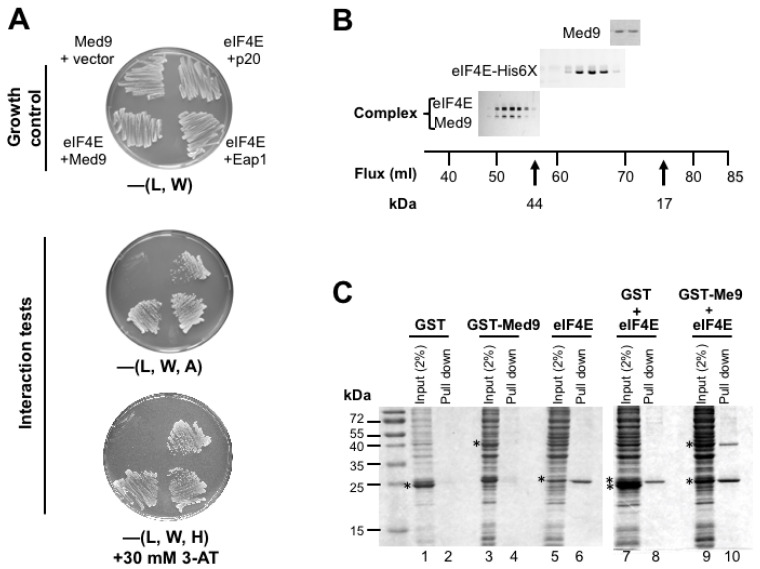
Med9 physically interacts with eIF4E. (**A**) Interaction in the yeast two-hybrid system. “Bait” eIF4E is in the binding domain; “preys” Med9, p20, and Eap1 are in the activator domain. Auxotrophic gene markers *Leu2*, *Trip1* and *His3*, and *Ade*, are used to grow in media lacking leucine (*L*), tryptophan (*W*), histidine (*H*), and adenine (*a*), respectively. Cells co-expressing both bait and preys were grown in –(*L*, *W*) medium. To detect interaction between baits and preys, cells were replated onto media –(*L*, *W*, *H*) + 30 mM 3-amino-1,2,4-triazole (*3AT*) and –(*L*, *W*, *A*). (**B**) Gel filtration profiles of Med9, eIF4E-His6X or Med9–eIF4E-His6X complexes using a S75 size exclusion column. Isolated recombinant Med9 (2–10 mg/mL) and eIF4E-His6X (2–10 mg/mL) were mixed in equimolar amounts, subjected to chromatographic separation on a S75 size exclusion column. The eluted fractions were resolved in SDS-PAGE and detected by silver staining. Med9 alone and eIF4E-His6X alone are purified at 18 kDa and 26 kDa, respectively. Together, they purified forming a complex. (**C**) m^7^GTP-sepharose pull-down assays using total protein extracts of bacteria expressing GST, GST-Med9, eIF4E or the indicated coexpressed proteins. Proteins were resolved in SDS-PAGE and detected by Coomassie staining. Asterisks point the recombinant proteins. GST-Med9, but not GST, co-pulled down with eIF4E. Input was 2%; pull down was 100%. ** represents two separate asterisks.

We rationalized that in organisms with a single eIF4E ortholog interaction with different, unidentified proteins are expected. Thus, novel eIF4E-interactors might fulfill the diverse functions performed by eIF4E paralogs in other species. To search for new eIF4E interactors in *S. cerevisiae*, we cloned eIF4E as a bait into the vector pOBD2 and performed a high-throughput yeast two-hybrid screen using an activation-domain library [37] in which two *S. cerevisiae* genomes were tested, i.e., 10,000–12,000 prey clones. To rule out false positives, we performed several rounds of cell growth in selective medium (lacking both tryptophan and leucine) at high stringency (i.e., containing 30 mM 3-amino-1,2,4-triazole, 3AT). We recovered seven clones encoding positive eIF4E interactors, namely p20, Eap1, ATPase-stabilizing factor family protein (STF2), histone chaperone SPT2, essential non-ATPase regulatory subunit of the 26S proteasome lid RPN3, myosin light chain 2 (MLC2), and Med9, a component of the RNA polymerase II mediator complex. Among them, Med9 consistently showed the strongest interaction with eIF4E. To corroborate this interaction, we PCR-amplified the Med9 cDNA from an independent, wild-type strain and performed two-hybrid assays again. As positive interactors, we used p20 and Eap1 cloned as preys onto the pOAD vectors (Figure 1A). Again, we observed clear interactions of eIF4E with Med9, p20 and Eap1, but not of Med9 with the empty vector (negative control).

We next corroborated the eIF4E–Med9 interaction using recombinant proteins. We cloned Med9 and eIF4E as GST and His6X tag fusions, respectively, and expressed the proteins in the bacterium *E. coli*. The proteins were purified and the GST tag was removed from Med9 using the PreScission protease cleavage. Pure Med9 and eIF4E-His6X proteins were analyzed by gel filtration FPLC through a size exclusion S75 column. Individual Med9 and eIF4E-His6X eluted to the expected sizes, namely 18 kDa and 25 kDa, respectively. When both proteins were mixed in equimolar amounts, they underwent a shift in the elution profile and eluted as a complex (Figure 1B). We next conducted m^7^GTP-Sepharose pull-down assays using the recombinant proteins GST-Med9 and eIF4E (Figure 1C). Whereas GST (lane 2) and GST-Med9 (lane 4) did not bind the resin, and GST did not copurify together with eIF4E (lane 8), GST-Med9 copurified along with eIF4E (lane 10), indicating a direct interaction.

### 2.2. Med9 and eIF4E Interact in Glucose and Galactose but Not in Lactate

**Figure 2:**
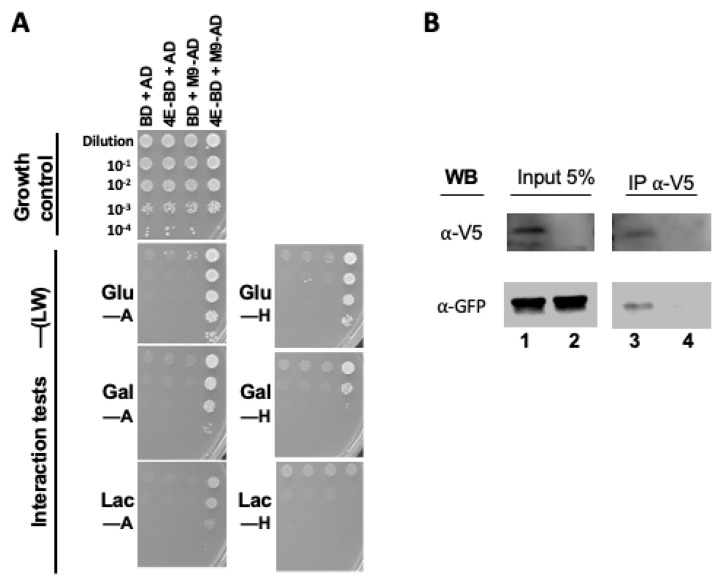
The formation of the eIF4E–Med9 complex depends on the carbon source. (**A**) Yeast two-hybrid system for serial dilutions of the indicated interactions. Cells were grown in the indicated carbon sources. *BD*, binding domain; *AD*, activator domain; *Glu*, glucose; *Gal*, galactose; *Lac*, lactate. –(*L*, *W*), growth control; –(*L*, *W*, *A*) and –(*L*, *W*, *H*), interaction tests. (**B**) Co-immunoprecipitation experiments using total cell lysates showing that Med9-V5 interacts with eIF4E-GFP in vivo. Lanes 1 and 2, input (5%). Lanes 3 and 4, co-IP using an anti-V5 epitope tag antibody. Lanes 1 and 3, strain *eIF4E-GFP <pVT-URA3-Med9-V5>*. Lanes 2 and 4, *eIF4E-GFP <pVT-URA3>* (empty vector, control). Western blots (*WB*) were performed using anti-V5-HRP and anti-GFP antibodies, as indicated.

In a high-throughput analysis of protein complexes isolated by co-immunoprecipitation assays, Ho et al. [38] purified a complex containing Med9, eIF4E, porin1, GLK1, and GMP1 when cells were grown in galactose as carbon source. This complex was isolated in the presence of total RNA. Thus, we performed two-hybrid interactions in different carbon sources. We platted diploid cells onto glucose, galactose, or lactate (Figure 2A) and cells were tested for growth (indicating interaction) in the absence of adenine (–*A*) or absence of histidine containing 30 mM 3AT (–*H*, Figure 2A). Two independent transformations were tested with similar results. A strong Med9–eIF4E interaction occurred in cells grown in glucose and galactose. A much weaker interaction was observed for lactate in the absence of adenine or the lack of interaction in the media minus histidine, showing dependence on the carbon source.

Next, we determined whether the endogenous proteins form a complex. According to mass spectrometry analyses, eIF4E is in vast excess over Med9, with estimated concentration of 36,000 eIF4E and 925 Med9 molecules per cell [39]. In addition, Med9 must displace eIF4G1, eIF4G2, Eap1, and p20 to bind eIF4E. Thus, we studied the yeast strain *CDC33-GFP*, which expresses eIF4E fused to the green fluorescent protein (eIF4E-GFP) under its endogenous promoter [40,41]. This strain was transformed either with the empty vector pVT-URA3 or the construct pVT-URA3-Med9-V5, which expresses Med9 in fusion with the V5 epitope tag under the control of the constitutive promoter *ADH1*. We grew the strains in a medium containing galactose as a carbon source and performed co-immunoprecipitation (co-IP) experiments of total extracts of *CDC33-GFP <pVT-URA3-Med9-V5>* and *CDC33-GFP <pVT-URA3>* (empty vector, control) using an anti-V5 antibody. For each co-IP, 5% of total extract was loaded as input (Figure 2B, lanes 1 and 2). As shown in Figure 2B, lane 3, eIF4E-GFP coimmunoprecipitated with Med9-V5 but not with the strain carrying the empty vector (Figure 2B, lane 4). Altogether, these experiments demonstrated a physical and stable interaction between Med9 and eIF4E that actually occurs in vivo.

Several eIF4E interactions with specific 4E-IPs are conserved in different species, including 4E-BPs and 4E-T, but are not in other cases such as Bicoid or Maskin [42,43]. Thus, we asked whether the Med9–eIF4E complex forms in species phylogenetically close to *S. cerevisiae*. We observed interaction in *S. kudriavzevii* (Supplementary Figure S1), the closest relative of *S. cerevisiae*. The *S. cerevisiae* eIF4E V71 and W75 involved in eIF4G1, p20, and Eap1 binding [20,44], and the Med9 residues contacting eIF4E, are also present in the *S. kudriavzevii* orthologs.

### 2.3. Med9 Possesses an eIF4E-Binding Site

**Figure 3:**
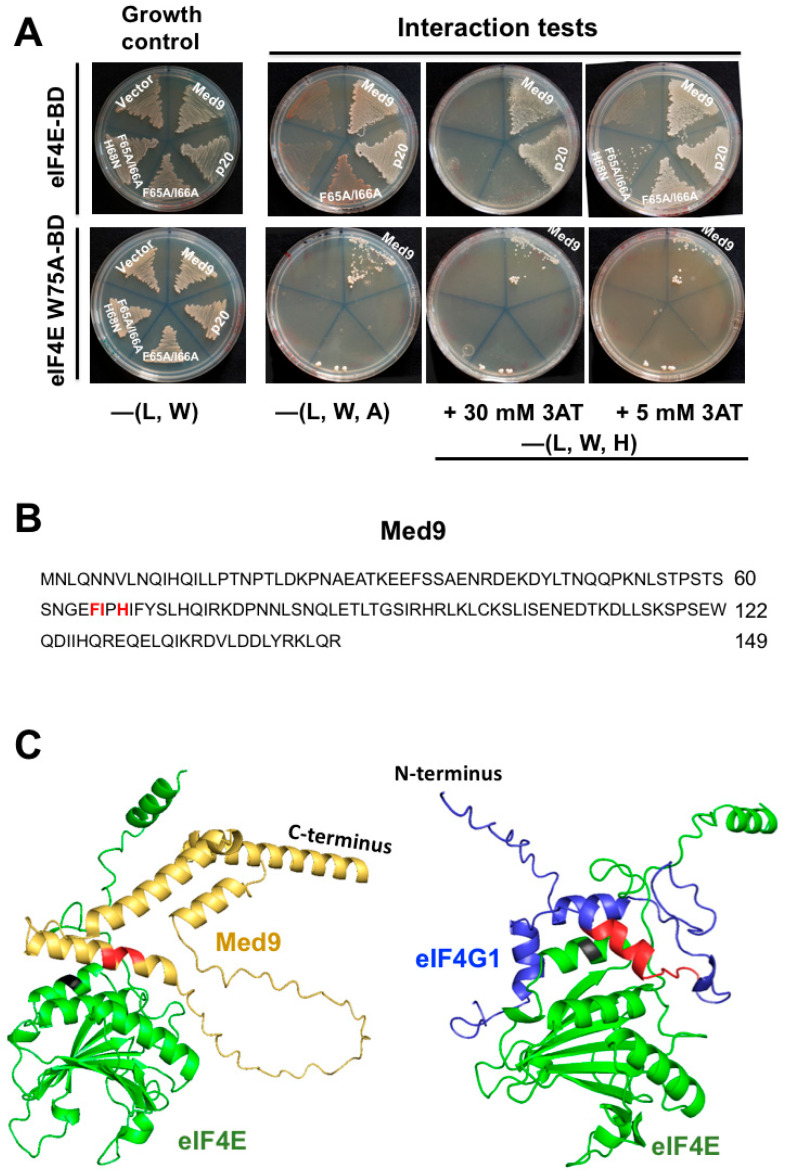
Med9 possesses a non-canonical eIF4E-binding site. (**A**) Interactions between wild-type eIF4E and the eIF4E W75A mutant with wild-type and Med9 mutants in the yeast two-hybrid system. eIF4E and eIF4E W75A mutant are expressed in the binding domain (BD). Wild-type Med9 and the mutants 65FA/I66A/H68N and 65FA/I66A, p20 (positive control) and empty vector (negative control) were expressed in the activator domain (AD). –(*L*, *W*), growth control; –(*L*, *W*, *A*), and –(*L*, *W*, *H*), interaction tests. *L*, leucine; *W*, tryptophan; *H*, histidine; *A*, adenine; *3AT*, 3-amino-1,2,4-triazole. (**B**) Sequence of the Med9 protein. The region 65FIPH68 when mutated towards 65AAPN68 failed to interact with eIF4E, and is highlighted in red. (**C**) AlphaFold3 prediction of interaction between eIF4E (green) and Med9 (yellow) (left) or eIF4E and eIF4G1 (amino acids 393–490) (blue) (right). NMR studies [44] described a sole eIF4G1 motif contacting eIF4E, whereas SAXS and co-IP experiments described two motifs [20]. AlphaFold3 modeling consists of two eIF4E-binding motifs present in eIF4G1. The eIF4E W75 residue that interacts with eIF4G1 and p20 [20,44] is depicted in black. The eIF4E-interacting site of Med9 (amino acids 65–68) and eIF4G1 (amino acids 449–461) is shown in red.

Multiple proteins contact different regions of the eIF4E dorsal surface. Most of them share the canonical eIF4E-binding motif YXXXXLϕ (where ϕ is Leu, M, or Phe) [4,25] and others interact via non-conserved binding sites [43,45,46]. Metazoan eIF4G and eIF4E-binding proteins (4E-BPs) physically bind the fragment 68S/TVXXXW73 (human protein numbering) at the dorsal region of eIF4E, where W73 is critical for this interaction [4,47,48]. A second, non-conserved eIF4E-binding site present in eIF4G and 4E-BPs wraps the lateral surface of eIF4E [20,49,50,51,52,53]. In yeast, eIF4E Val71 and W75 (equivalent to human W73), which are located on the convex dorsum of the protein, contact eIF4G1, p20, and Eap1 [20,44]. Thus, we asked what region of Med9 interacts with eIF4E. We randomly mutated the Med9 cDNA and further performed yeast two-hybrid assays to test interactions between wild-type (Figure 3A, upper) or a W75A mutant eIF4E (Figure 3A, lower), with wild-type Med9 and the randomly generated Med9 mutants. We also tested p20 as a positive control. Among approximately 80 Med9 mutants, we observed that the mutations F65A/I166A and F65A/I66A/H68N did not interact with wild-type eIF4E nor eIF4E W75A. It is noteworthy that, while p20 (positive control) lost interaction with eIF4E W75A, cells expressing wild-type Med9 still showed growth with this mutant, suggesting a stronger interaction with eIF4E’s dorsal surface. The Med9 mutations are shown in Figure 3B.

We further ran in silico AlphaFold3 [54] docking using the published three-dimensional protein structures as templates [20,44,55]. AlphaFold3 modeling was consistent with the experimentally supported interaction, and predicted the formation of a Med9–eIF4E complex via the analyzed amino acids (Figure 3C, left), whereas modeling utilizing the mutants eIF4E W75A and Med9 F65A/I66A/H68N suggested no interaction of the same regions of the proteins, in agreement with the two-hybrid observations. Our mutagenesis and AlphaFold3 docking results suggest that the Med9 region 65FIPH68 located on the α1 helix of the protein interacts with the dorsal surface of eIF4E. For comparison, we also ran molecular docking between eIF4E and an eIF4G1 fragment (amino acids 393–490) containing the eIF4E-binding site (Figure 3C, right). Whereas Gross et al. (2003) [44] found only a single eIF4E-binding site present in eIF4G1 as determined by NMR experiments, further experiments by the Izaurralde group using co-immunoprecipitation (co-IP) and small-angle X-ray scattering (SAXS) demonstrated that eIF4G1 also possesses a second, non-canonical eIF4E-binding motif 14 amino acids away from the first one that contacts the lateral surface of eIF4E [20]. The AlphaFold3 modeling agreed with the eIF4E–eIF4G interaction via two binding sites (Figure 3C, right).

### 2.4. eIF4E and Med9 May Localize in Cytoplasm and Nucleus

**Figure 4:**
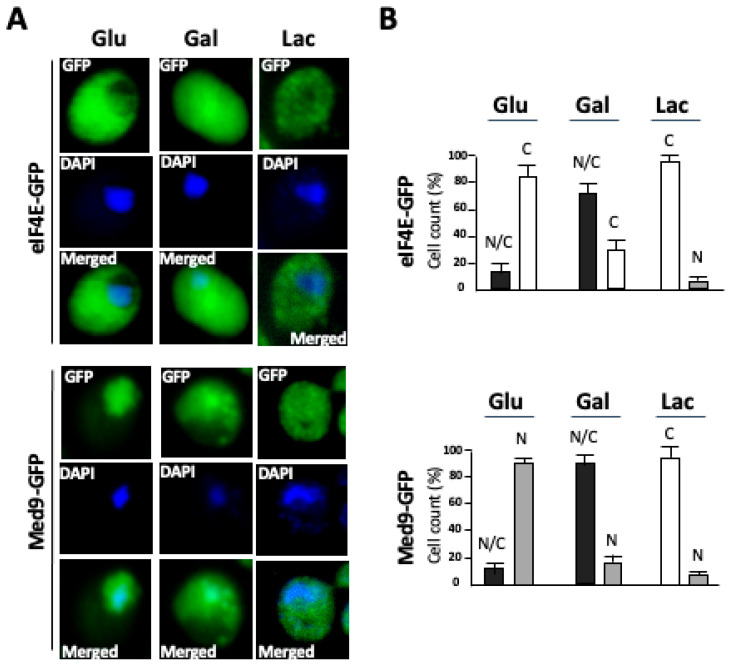
The localization of eIF4E and Med9 depends on the carbon source. (**A**) Cellular distribution of eIF4E-GFP and Med9-GFP grown in glucose, galactose, or lactate as carbon sources. Epifluorescence images are shown. Magnification = 100×. (**B**) Cell count of the indicated cellular distribution of the protein. N/C, nuclear and cytoplasmic localization; C, cytoplasmic localization; N, nuclear localization. The percentage of cells showing the indicated distribution is indicated by the bars.

We next analyzed protein localization using the *CDC33-GFP* and *CSE2-GFP* strains encoding eIF4E and Med9 fused to GFP (eIF4E-GFP and Med9-GFP, respectively) [40,41]. We grew the cells in glucose or galactose as a carbon source and counted the percentage of cells showing a specific protein distribution, i.e., nuclear, cytoplasmic or both. In all cases, we did not find 100% of the cells with a specific protein distribution, but rather a mixture of cells with different cellular distribution of the indicated proteins. We observed a significant shift in the percentage of cells with a cellular distribution of proteins depending on the medium used (Figure 4A, upper). In glucose, 80% of the cells showed mostly cytoplasmic eIF4E-GFP and about 20% of the cells showed a nuclear and cytoplasmic localization. In contrast, 80% of the cells showed a nuclear and cytoplasmic localization when grown in galactose, indicating an eIF4E-GFP import toward the nucleus (Figure 4B, upper). In glucose, 90% of the cells had Med9-GFP distributed mostly in the nuclear (Figure 4A, lower), but became distributed in both the nuclear and cytoplasm in 95% of the cells when grown in galactose (Figure 4B, lower). When we grew the cells in lactate, both eIF4E-GFP and Med9-GFP showed mostly a cytoplasmic localization (Figure 4B). Altogether, these results suggest that the endogenous Med9–eIF4E complex might be formed both in the cytoplasm and within the nucleus when grown in glucose and galactose, but not lactate.

## 3. Discussion

### 3.1. Transcription Factor Med9 Is a Novel eIF4E Partner

In a two-hybrid genomic screen, we identified Med9 to be a novel interactor of eIF4E and further corroborated its physical and endogenous interaction. This interaction is conserved in the closely related yeast *S. kudriavzevii*. Our discovery of the Med9–eIF4E complex agrees with the finding of a protein complex isolated by Ho et al. [38] containing Med9 and eIF4E (among other proteins) from cells grown in galactose as a carbon source.

Med9 is a component of the RNA polymerase II transcriptional Mediator complex [56,57,58]. Interestingly, other transcription factors interact with eIF4E orthologs across species. This is the case of human HOX9 [8] and PRH [59], mouse EMX2 [60] and PREP1 [61], and *Drosophila* Bicoid [62]. Thus, eIF4E interaction with transcription factors to regulate gene expression is a recurrent phenomenon that has evolved independently several times in different lineages [42].

### 3.2. Med9 Possesses a Non-Canonical eIF4E-Binding Site

Metazoan proteins bind eIF4E via canonical (YXXXXLϕ) [4,25] and non-conserved motifs that recognize the dorsal and lateral surfaces of eIF4E, respectively, in a bipartite interaction mode [20,49,50,51,52,53]. This is also the case for the yeast eIF4G1 and Eap1 [20]. We described that the Med9 region contacting eIF4E is rather different than the canonical motif. AlphaFold3 in silico docking predicted a non-bipartite mode of interaction between Med9 and eIF4E.

### 3.3. eIF4E–Med9 Complex Forms in Galactose and Glucose

*S. cerevisiae* ideally grows in media containing the fermentable sugars glucose or galactose. Both carbon sources induce the expression of genes in the glycolytic or galactose pathways [63]. A shift to a non-fermentable, lactate-containing medium drives respiratory reprograming to obtain energy via oxidative phosphorylation and the activation of genes involved in gluconeogenesis [64]. Med9 is less abundant in the cell than eIF4E [39], and *CSE2* expression does not seem to be significantly affected when yeast cells are grown in fermentative or respiratory media [65], indicating that the observed interaction in fermentative media is not due to increased Med9 concentrations. Further experiments should be done to elucidate the biological relevance of this complex in gene expression during growth in different carbon sources.

### 3.4. Possible Nuclear Complex eIF4E–Med9

Protein localization suggests that the eIF4E–Med9 complex might be formed in both the cytoplasm and inside the nucleus. In the cytoplasm, Med9 must compete with other eIF4E-interacting proteins for eIF4E binding. Moreover, eIF4E is in vast excess over Med9 [39]. Thus, Med9 is unlikely to affect translation. Instead, eIF4E could easily have an effect on the Med9-mediated transcription in the nucleus. Indeed, the two-hybrid interactions occur within the nucleus. In monkey, human, *Drosophila*, and mouse cells [8,66,67,68,69,70,71,72], a fraction of eIF4E lies within the nucleus in complex with several interactors to mediate mRNA export or 3′ end pre-mRNA processing [9,73]. In other systems, including *Xenopus* [74], *Giardia* [75], *Trypanosoma brucei* [76,77], and *S. cerevisiae* [78] (this study), eIF4E orthologs have also been detected in the nucleus, but their role there in these species is unknown. While the Med9–eIF4E complex could be formed in the cytoplasm, we suggest that a fraction of this complex also exists within the nucleus perhaps to mediate transcription. Further experimentation should be performed to investigate this notion.

## 4. Material and Methods

### 4.1. Culture, Manipulation and Molecular Biology of Yeast

Protocols for yeast growth and manipulation, and yeast DNA and proteins isolation were from Xiao (2014) and Nosek and Thomaska (2013) [79,80].

### 4.2. Plasmid Construction

DNA manipulation, PCR-amplification, cloning and sequencing were done according to Green and Sambrook (2012) [81]. PCR-amplification of DNA fragments was carried out using Taq DNA Polymerase (ThermoFisher, Waltham, MA, USA) and specific oligonucleotides. We cloned *S. cerevisiae* eIF4E (gene ID: *CDC33*) and Med9 (gene ID: *CSE2*) into the pGEX-6P2 (Amersham) and pET-28 or pET-30 (Novagen, Madison, WI, USA) vectors in-frame with the glutathione S-transferase (GST) or histidine (His6X) tags, respectively. The cDNAs were also cloned into the pRSET vector (ThermoFisher) without any tag. Different versions of *S. cerevisiae* eIF4E and Med9 cDNAs were subcloned onto the two-hybrid vectors pOAD and or pOBD2 [82] in-frame either with the activator domain (AD) or the DNA-binding domain (BD) sequence of GAL4, respectively, to create the “prey” plasmids eIF4E-AD and Med9-AD, and the “bait” plasmids eIF4E-BD and Med9-BD. For immunoprecipitation experiments, the Med9 cDNA was subcloned in-frame with the V5 epitope tag (GKPIPNPLLGLDST) onto the pVT-URA3-102 vector under the constitutive *alcohol dehydrogenase* promoter (*ADH*) [83]. Mutations in the Med9 and eIF4E cDNAs were generated using the QuikChange Site-Directed Mutagenesis kit (Stratagene, La Jolla, CA, USA) and further subcloned onto the indicated plasmids.

### 4.3. High-Throughput Screens and Interaction Assays in Yeast Two-Hybrid System

We performed a high-throughput yeast two-hybrid screen using the eIF4E-binding domain (BD) construct as a “bait” against an activation-domain yeast library [37] according to [82]. Cells were transformed using the lithium acetate-polyethylene glycol method [84]. We screened approximately 12,000 clones of *S. cerevisiae* genes in the plasmid pOAD (prey, activator domain), representing two genomes, and platted onto thirty-two 384-well microtiter plates of –Leu medium using a robotic replicating tool (Biomek 2000, Beckman Coulter, Brea, CA, USA) [82]. Interactions between “bait” and “prey” proteins were detected following an interaction-mating method using the strains PJ69-4α [85] transformed with “baits” and PJ69-4a [86] transformed with “preys” containing the reporter genes *HIS3* and *ADE2* [82]. Diploid cells containing both bait and prey plasmids were grown in –(Trp, Leu) selective media (Clontech, Mountain View, CA, USA) and shown as controls for growth. Protein interactions were detected by replica-plating diploid cells onto –(Trp, Leu, Ade) or –(Trp, Leu, His) selective media (Clontech) containing either 5 mM or 30 mM 3-amino-1,2,4-triazole (3AT, Sigma-Aldrich, St. Louis, MO, USA). Growth was scored after 4–5 days of growth at 30 °C.

### 4.4. Production of Recombinant Proteins

The GST Gene Fusion System from Amersham and the QIAexpressionist (Qiagen, Hilden, Germany) protocols were used to produce and purify recombinant protein. Briefly, *E. coli* BL21 (DE3) One shot (Invitrogen, Carlsbad, CA, USA) transformed with the plasmids pGEXT-eIF4E, peIF4E-His6X, pGEXT-Med9, or pMed9-His6X, and were cultured and induced with 0.5 mM IPTG for 3 h at 37 °C with shaking at 200 rpm. The cultures were centrifuged, and the pellets treated with lysozyme followed by French press cell disruption in the presence of Complete Protease Inhibitor Cocktail EDTA-free (Roche, Basel, Switzerland). The resulting supernatant, containing solubilized GST-eIF4E, eIF4E-His6X, GST-Med9, or Med9-His6X were incubated with Glutathione-Sepharose 4B (GST-eIF4E and GST-Med9) or Ni-NTA-Agarose Superflow (Qiagen) (Med9-His6X and eIF4E-His6X) resins for 45 min at 4 °C. Subsequently, the sepharose was recovered and washed once with PBS. GST-eIF4E and GST-Med9 were cleavaged out using PreScission Protease (Merck, Darmstadt, Germany) for 4 h at 4 °C. Following cleavage, the supernatant containing eIF4E or Med9 was recovered.

### 4.5. FPLC Analysis

Fast protein liquid chromatography (FPLC) was performed according to Schütz et al., 2008 [87]. Two to ten micrograms/mL of recombinant proteins were diluted in column buffer (20 mM Tris-HCl pH 7.5, 100 mM NaCl, 1 mM EDTA) and further passed through a Superdex 75 (S75) gel filtration column (Amersham Pharmacia, Uppsala, Sweden). The collected fractions were analyzed in SDS-PAGE and silver staining.

### 4.6. m^7^GTP-Sepharose Pull Down

A total of 50 mL of bacterial cultures were grown 3 h at 37 °C and then induced for 3 h with 1 mM IPTG, further pelleted and resuspended in 2.5 mL cap-binding buffer (CBB; 100 mM KCL, 20 mM HEPES pH 7.6, 0.2 mM EDTA, 10% glycerol, 0.1% Triton X100, Complete Protease Inhibitor Cocktail EDTA-free, 0.2 mM PMSF, 50 mM beta-glycerol phosphate, 7 mM beta-Mercapto ethanol, and 1 mM orthovanadate) on ice. Cells were lysed with lysozyme and sonication on ice. Lysates were pelleted and 1 mL supernatant was pre-cleared with 150 µL Sepharose CL 4B (GE Healthcare, Uppsala, Sweden) preequilibrated 1 h in CBB with gentle rotation at 4 °C. Beads were removed by centrifugation for 2 min at 2500 rpm. Lysates were further incubated either with 150 µL slurry of m^7^GTP-Sepharose 4B beads (GE Healthcare) or Sepharose CL 4B (control) pre-equilibrated in CBB for 1 h at 4 °C with gentle rotation. The beads were collected by gravity on ice, washed three times with 750 µL of CBB containing 1 mM GTP. Subsequently, the beads were recovered and resuspended in SDS-PAGE sample buffer and subjected to SDS-PAGE and Coomassie staining.

### 4.7. Fluorescence Microscopy

The *S. cerevisiae* strains *CDC33-GFP* and *CSE2-GFP* [40,41] (https://yeastgfp.yeastgenome.org/ accessed on 1 December 2025) encoding eIF4E-GFP and Med9-GFP, respectively, were grown to OD_600_ = 1.0 in the indicated media containing glucose, galactose, or lactate (Sigma) and imaged as described in [40] using Zeiss Axioskop 2 mot plus fluorescence microscope (Carl Zeiss, Oberkochen, Germany), and a 100× objective with Zeiss axiocam MRc5 using Zen 3.4 software. Fluorescence quantitation of fluorescent proteins was performed using the Fiji (Image J, Version 1.54, 2025) algorithm [88].

### 4.8. Co-Immunoprecipitation Assays

*CDC33-GFP* cells [40,41] were transformed with the plasmid pVT-URA3-102-Med9-V5 or pVT-URA3-102 (empty vector, control) and selected in medium YNB, glucose 2%, and dropout-uracil (Clontech). *CDC33-GFP* <*pVT-URA3-102-Med9-V5*> and *CDC33-GFP* <*pVT-URA3-102*> cells were grown in YNB, galactose 2%, and dropout-uracil to OD_600_ = 1.0, harvested, resuspended in 1 mL distilled and sterile water, and further lysed in immunoprecipitation (IP) buffer (30 mM HEPES pH 7.4, 100 mM KOAc, 2 mM Mg(OAC)_2_, fresh 2 mM DTT, and mini Complete EDTA-free protease inhibitor cocktail) by bead-beating precooled acid-washed glass beads and 30 s vortex pulses. Cell lysates were spun for 10 min at 2300 rpm at 4 °C, and the supernatant was quantified for total protein concentration and stored at −80 °C.

Total protein was precleared for 1 h at 4 °C with 200 L of 50% slurry of Protein-A-Sepharose beads (4 Fast Flow; GE Healthcare) equilibrated with IP buffer. 500 g of total lysate was co-immunoprecipitated with gentle rotation for 2 h using 3.5 L of anti-V5 antibody (Santa Cruz, Dallas, TX, USA) and 40 L of the 50% slurry of Protein-A-Sepharose beads equilibrated with IP buffer. The beads were washed 2 times for 20 min with 500 L IP buffer, and centrifuged for 1 min at 2300 rpm at 4 °C. A final wash was done with 500 L of Tris-HCl 20 mM pH 7.4 and centrifuged for 1 min at 2300 rpm at 4 °C. The supernatant was completely discarded using a micro syringe (Hamilton, Reno, NV, USA). Afterward, Laemli sample buffer was added to the beads, which were boiled for 5 min and analyzed by SDS-PAGE and immunoblotting using anti-V5-horse radish peroxidase (HRP) monoclonal conjugated primary antibodies (anti-V5-HRP; Sigma), 1:5000 or mouse anti-GFP primary antibodies (Santa Cruz), 1:2000. HRP-conjugated goat anti-mouse (1:5000) secondary antibodies (GE Healthcare) and ECL enhanced chemiluminescence reagent (Perkin Elmer, Shelton, CT, USA) were used to detect anti-GFP antibodies. To detect anti-V5 antibody, Immobilon Western chemiluminescent HRP substrate (Millipore, Burlington, MA, USA) was used.

### 4.9. Protein Docking

Protein–protein docking was predicted by AlphaFold3 [54] using the Worldwide Protein Data Bank (PDB) 3D structures of *S. cerevisiae* eIF4E (code 6FC1 [20]), Med9 (code 1YKH [55]), and eIF4G1 (amino acids 393–490) (code 1RF8 [44]). Images were visualized using PyMOL software, Version 2.6, 2025; PyMOL retrieved from http://www.pymol.org/pymol (accessed on 1 December 2025).

### 4.10. Sequences Alignment

Protein accession numbers are the following: *S. cerevisiae* eIF4E (ID: NP_014502.1) and Med9 (ID: P33308); *Z. rouxii* eIF4E (ID: C5DSV2) and Med9 (ID: C5DT46); *S. kudriavzevii* eIF4E (ID: JGEGQ4) and Med9 (ID: JGECA8); and *C. glabrata* eIF4E (ID: Q9P974) and Med9 (ID: Q6FQJ5). Sequences were obtained from UniProt.org (https://www.uniprot.org/) and OrthoDB.org (https://www.orthodb.org/), aligned using the Clustal Omega program, Version 1.2.4 (https://www.ebi.ac.uk/Tools/msa/clustalo/ (accessed on 20 November 2025)), and optimized by eye. The following conservative amino acids were considered: G and A; S and T; K, R and H; E, D, Q and N; and L, I, M, V, C, Y, F, and W.

## Data Availability

All data and results of this work are in the article and in Supplemental Materials.

## Supplementary Materials

The following supporting information can be downloaded at https://www.mdpi.com/article/10.3390/ijms27156911/s1.
