# Supplementary material for: Med9 Is a Novel eIF4E-Interacting Protein in Saccharomyces cerevisiae
Source: Int J Mol Sci. 2026 Aug 1;27(15):6911. doi: 10.3390/ijms27156911 (PMC13467561; doi:10.3390/ijms27156911)
Supplement: Supplementary file 1 [file ijms-27-06911-s001.zip › ijms-4334662-supplementary.pdf]

Supplementary Material for:

Med9 interacts with eIF4E dependent on the carbon source in *Saccharomyces cerevisiae*

Dora E. Vélez[1], Zorica Ristic[2§], Daniela Ross-Kaschitza[2], Michael Altmann[2], Angélica Montiel-Dávalos[1], Vincent G. Osnaya[1], Yolanda Camacho-Villasana[3], Xochitl Pérez-Martínez[3] and Greco Hernández[1, 2, 4*]

1) mRNA and Cancer Laboratory, Subdirección de Investigación Básica, National Institute of Cancer (Instituto Nacional de Cancerología, INCan). 14080-Mexico City, Mexico.
2) Institut für Biochemie und Molekulare Medizin (IBMM), Universität Bern. 3012-Bern, Switzerland.
3) Cell Physiology Institute (Instituto de Fisiología Celular), Universidad Nacional Autónoma de México, 04510-Mexico City, Mexico.
4) Tecnologico de Monterrey, Escuela de Medicina y Ciencias de la Salud. Mexico.
§) Present address: ETH Phenomic Center, ETH Zürich, Zürich, Switzerland.

*Corresponding author: Greco Hernández

Email: ghernandezr@incan.edu.mx; greco.hernandez@tec.mx

Phone: +52/55/3693 5200 ext. 246

The Med9—eIF4E complex is conserved in *Saccharomyces kudriavzevii*

We cloned the ortholog proteins from *S. kudriavzevii*, *Zygosaccharomyces rouxii* and *Candida glabrata* (Suppl. Fig. 1A), all belonging to the ascomycetal Family *Saccharomycetaceae*, and assayed interaction in the two-hybrid system. We observed interaction in *S. kudriavzevii* (Suppl. Fig. 1B), which is the closest relative species to *S. cerevisiae*, but not in *C. glabrata* or *Z. rouxii*. The *S. cerevisiae* V71 and W75 eIF4E involved in eIF4G1, p20, and EAP1 interaction, and the Med9 residues contacting eIF4E, are also present in the *S. kudriavzevii* orthologs.

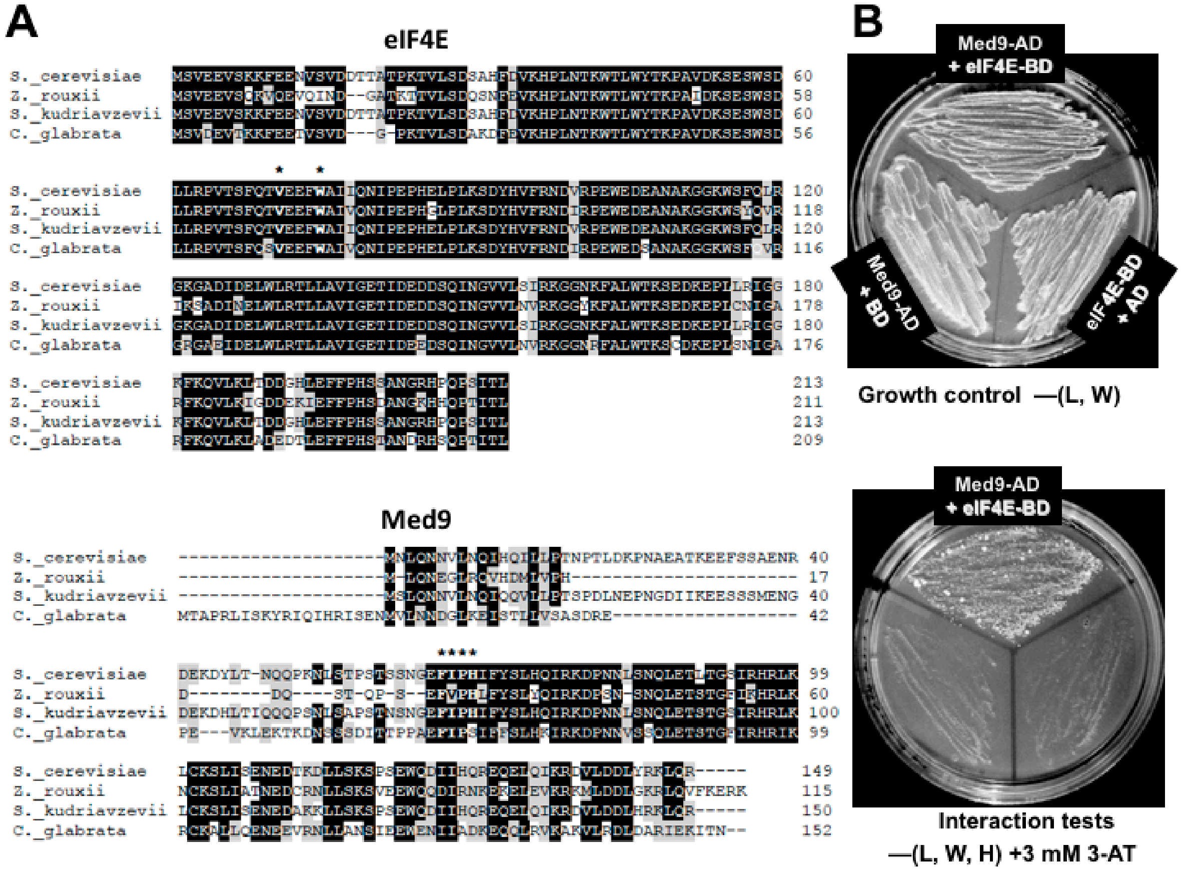

Supplemental Figure S1. The interaction Med9—eIF4E is conserved in the yeast *Saccharomyces kudriavzevii. A)* Sequence comparison of full-length eIF4E (*left*) and Med9 (*right*) of the indicated species of the fungal Family *Saccharomycetaceae*. The V71 and W75 of *S. cerevisiae* eIF4E involved in eIF4G1, p20, and EAP1 interaction, and the *S. cerevisiae* Med9 residues contacting eIF4E, are indicated by *Asterisks*. Residues identical in at least three sequences are

shaded in black boxes. Conservative changes in at least three sequences are in grey boxes. Gaps are represented by dashes. ***B)*** Interaction assay in the two-hybrid system. Only *S. kudriavzevii* Med9—eIF4E interaction is shown. *Upper*, growth control. *Lower*, interaction test. Interaction with empty vectors was assayed as negative control. *AD*, activator domain; *BD*, binding domain.

Supplemental Material and Methods

Plasmid construction and yeast two-hybrid assays

Med9 from the ascomycetes *Zygosaccharomyces rouxii* (gene ID: *ZYRO0C05412g*), *Saccharomyces kudriavzevii* (gene ID: *YNR010W*), and *Candida glabrata* (gene ID: *CAGL0I05742g*) were PCR-amplified using genomic DNA as template and further cloned in-frame with the GAL4 activator domain to create the respective “prey” plasmids. eIF4E from *Z. rouxii* (gene ID: *ZYRO0C03234g*), *S. kudriavzevii* (gene ID: *YOL139C*), and *C. glabrata* (gene ID: *CAGL0E01463g*) were PCR amplified using genomic DNA as template and further cloned in-frame with the GAL4 DNA-binding domain sequence of GAL4 to create the respective “bait” plasmids. The genomic DNA from *Z. rouxii* (cat. num. 2623D-5) and *S. kudriiavzevii* (cat. num. MYA-4449D-5) were purchased from ATCC. The genomic DNA from *C. glabrata* was a kind gift of Irene Castaño (IPICyT, Mexico).
